# Supplementary material for: The Subcutaneous Administration of Beta-Lactams: A Case Report and Literary Review—To Do Small Things in a Great Way
Source: Infect Dis Rep. 2024 Jan 29;16(1):93–104. doi: 10.3390/idr16010007 (PMC10887887; doi:10.3390/idr16010007)
Supplement: Supplementary file 1 [file idr-16-00007-s001.zip › idr-2814241-supplementary.pdf]

Search strategy:

Pubmed: ("beta-lactams"[All Fields] OR "beta-lactams"[MeSH Terms] OR beta-lactams[Text Word] OR "Carbapenems"[Mesh] OR "Cephalosporins"[Mesh] OR "Monobactams"[Mesh] OR "Penicillins"[Mesh]) AND ("infusions, subcutaneous"[MeSH Terms] OR "injections, subcutaneous"[MeSH Terms] OR "subcutaneous administration"[text word]

Scopus: (beta-lactams OR beta-lactams OR beta-lactams OR Carbapenems OR Cephalosporins OR Monobactams OR Penicillins) AND ("infusions, subcutaneous" OR "injections, subcutaneous" OR "subcutaneous administration")

Web of Science: (beta-lactams OR beta-lactams OR beta-lactams OR Carbapenems OR Cephalosporins OR Monobactams OR Penicillins) AND ("infusions, subcutaneous" OR "injections, subcutaneous" OR "subcutaneous administration")
